# Unraveling the genetic basis of methane emission in dairy cattle: a comprehensive exploration and breeding approach to lower methane emissions

**DOI:** 10.1080/10495398.2024.2362677

**Published:** 2024-06-11

**Authors:** Destaw Worku

**Affiliations:** Department of Animal Science, College of Agriculture, Food and Climate Science, Injibara University, Injibara, Ethiopia

**Keywords:** Carbon foot print, CH_4_ emission, dairy cattle, genomic regions, breeding strategies

## Abstract

Ruminant animals, such as dairy cattle, produce CH_4_, which contributes to global warming emissions and reduces dietary energy for the cows. While the carbon foot print of milk production varies based on production systems, milk yield and farm management practices, enteric fermentation, and manure management are major contributors togreenhouse gas emissions from dairy cattle. Recent emerging evidence has revealed the existence of genetic variation for CH_4_ emission traits among dairy cattle, suggests their potential inclusion in breeding goals and genetic selection programs. Advancements in high-throughput sequencing technologies and analytical techniques have enabled the identification of potential metabolic biomarkers, candidate genes, and SNPs linked to methane emissions. Indeed, this review critically examines our current understanding of carbon foot print in milk production, major emission sources, rumen microbial community and enteric fermentation, and the genetic architecture of methane emission traits in dairy cattle. It also emphasizes important implications for breeding strategies aimed at halting methane emissions through selective breeding, microbiome driven breeding, breeding for feed efficiency, and breeding by gene editing.

## Introduction

The world’s 1.4 billion cattle population both contributes to the agricultural greenhouse gas (GHG) emissions and suffers from high ambient temperatures combined with humidity.^1^^,^^2^ With the ongoing global population growth, increasing income levels, and urbanization, there is an anticipated rise in the demand for animal products. The projected increases in demand for various animal products necessitate a corresponding increase in production efficiency for animal species. Despite improvements in production efficiency, the commensurate increase in GHG emissions from livestock systems remains a significant challenge.^3^ The Intergovernmental Panel on Climate Change (IPCC) highlighted the substantial contribution of agriculture, predominantly by livestock, to the increase in atmospheric CH_4_ emissions.^4^ Among the livestock species, cattle are the primary contributor to GHG emissions, accounting for ∼62% of all livestock emissions.^3^ When considering emission sources within the livestock sector, the emissions of CH_4_ from enteric fermentation account for more than half (54%) of total (78%) livestock emissions, of which, cattle contribute approximately three-quarter (76%) of the GHG emissions from enteric fermentation as well as around one-third (33%) from manure management.^3^ Each cow generates between 70 and 120 kg of CH_4_ annually.^5^

The potential consequences of CH_4_ emissions from ruminants pose significant concerns for both environmental sustainability and energy efficiency in the dairy industry. To address this issue, various strategies have been proposed to reduce ruminant CH_4_ emissions, including adjusting management practices and implementing nutritional treatments.^6^ However, these approaches are usually not cost-effective for farmers and can have a significant impact on CH_4_ production, thus, finding an effective and sustainable solution to the problem remains challenging.^7^^,^^8^ Genetic selection for low CH_4_-emitting animals, particularly dairy cows, is proposed as the best mitigation strategy that is cost-effective, permanent, and cumulative across generations.^9–11^ However, the success of this approach relies on the existence of genetic variation in CH_4_ production and favorable genetic associations of CH_4_ traits with other breeding goal traits.^7^ Several studies have reported the genetic control of CH_4_ emission traits and the existence of sizable genetic variation in CH_4_ emission among dairy cattle.^9^^,^^12–16^ While, methane emission traits in dairy cattle, such as daily methane production, methane yield, and methane intensity, are heritable and can be targeted through genetic selection programs aimed at mitigating GHG emissions,^7^^,^^17^ it is crucial to acknowledge that multiple genes contribute to the phenotype, making it a complex trait. Consequently, genetic progress through conventional genetic selection methods is slow. Therefore, gaining a better understanding of the genetics involved in CH_4_ emissions can generate valuable insights for genomic prediction and achieve rapid genetic progress for reduction in CH_4_ emission in dairy cattle. This prompts the need to identify genomic variants associated with different CH_4_ emission traits, thus contributing an additional feature in the headway of genomic selection of low CH_4_ emitter dairy cows. In this regard, genome-wide association studies (GWAS) have identified several genomic regions and variants affecting CH_4_ emission traits in dairy cattle, thus providing an additional attribute to improve genetic gain in the process of genomic selection.^7^^,^^11^^,^^18^^,^^19^ Since genome-wide significant single nucleotide polymorphisms (SNPs) have become affordable, genomic selection has been tested and would be of great value for selecting low emitter dairy animals with a desirable genetic makeup, as they would facilitate the selection of true causal variants and genes controlling complex traits. However, the economic value of an identified quantitative trait nucleotide (QTN) in the selection program will depend on the effects of the QTN on the traits included in the selection index, gene interactions, interactions of the QTL with other genes, and the frequency of the QTL alleles in the commercial population.^20^ Indeed, this review aims to undertake a comprehensive search for the carbon foot print (CF) of milk production, major emission hotspots, rumen microbiota and enteric methane emissions, and methane reducing cow feed additives. Additionally, it explores the genetic basis of CH_4_ emission traits in dairy cattle. Given its significance for future breeding programs, a holistic breeding strategy to halt CH_4_ emissions in dairy cows are also discussed.

## Carbon footprint of milk production in dairy cattle

The carbon footprint (CF) is an environmental indicator, that measures the direct and indirect GHG emissions released into the atmosphere during the entire life cycle of a product within the production chain.^21^ It is typically expressed as kilogram of CO_2_ equivalent (CO_2_e) per kilograms of product.^22^ To develop effective GHG mitigation strategies for milk production, it is important to understand the CF in terms of CO_2_e per kilogram of fat and protein corrected milk (CO_2_e/kg FPCM) specific to different regions.

Several studies (Table 1) have suggested that the major GHG contributing to the CF of milk on a dairy farm are CH_4_ from enteric fermentation and nitrous oxide from manure management and feed production.^23–26^^,^^29^^,^^34^^,^^36^^,^^40^ It is worth mentioning that, the estimated emissions for producing 1 kg of fat and protein corrected milk (FPCM) amounted to 1.23 kg of CO_2_e, with enteric methane accounting for 46% and manure management accounting for 32.7% of these emissions.^23^ In countries where milk production is predominantly based on pasture systems, over 50% of the CF can be attributed to CH_4_ emissions from enteric fermentation.^29^ Gollnow et al.^39^ reported that the largest share of the CF comes from enteric fermentation (57%) and manure (9%) deposited directly into pastures during grazing. Furthermore, Jayasundara et al.^31^ claim that enteric fermentation (44%) and the production and supply of feed (36%) contribute the most to the CF of milk.

**Table 1.** Summary of recent studies on carbon footprint (CF) and milk production in dairy cattle.

Estimated emission (kg CO_2_e/kg FPCM)	Production systems	Major emission contributors or hotspots			Approach	Country	References
		1st	2nd	3rd			
1.23	Intensive	Enteric fermentation (46%)	Manure management (32.7%)	NA	LCA	South Dakota	23
1.13 1.24 1.52	Grazing Mixed and housed	Enteric fermentation (32–51%)	Concentrate production (housed: 29%, mixed: 24%, grazing: 13%)	Manure management (housed: 19%, mixed: 9, grazing: 5%)	LCA	Europe	24
1.45–1.81	Smallholder dairy farms	Enteric fermentation (66.8%)	Feed production (23%)	Manure management (<4.50%)	LCA	India	25
2.26	Silvo-pastoral	Enteric fermentation	Manure management	Land use and energy/transport	LCA	Peruvian Amazon	26
2.35	Smallholder dairy farms	Enteric fermentation (67%)	Manure management (16%)	NA	LCA	Ethiopia	27
0.99	Average Dutch dairy system	Enteric methane	Roughage production	Purchased resource	LCA	NL	28
2.11	Intensive	Enteric fermentation	Manure management	Feed production	Syst. Rev	Range of countries	29
0.75–0.81	Grazed pasture dairy system	Enteric CH_4_ from dairy cows (85–86%)	Enteric CH_4_ from replacement animals (12–13%)	Manure management (1–2%)	LCA	NZ	30
0.44–1.73	Intensive	Enteric fermentation (44%)	Feed production (36%)	NA	LCA	Canada	31
2.19 and 3.13	Smallholder	Enteric fermentation (55.5%)	Feed production and transport (31.6%)	Manure management (12.6%)	LCA	Kenya	32
1.75	Large-scale	Enteric fermentation and manure (58%)	Transport and processing of wheat bran (20%)	Cultivation of roughages (15%)	LCA	Ethiopia	33
2.25	Peri-urban	Enteric fermentation and manure (47%)	Transport and processing of wheat bran (18%)	Cultivation of roughages (19%)	LCA	Ethiopia	33
2.22	Rural farms	Enteric fermentation and manure (64%)	Transport and processing of wheat bran (9%)	Cultivation of roughages (22%)	LCA	Ethiopia	33
1.39	Pasture-based	Enteric fermentation	Manure management	Electricity	LCA	South Africa	34
0.89	Pasture-based	Enteric fermentation	Concentrate feed production	Fertilization	LCA	Portugal	35
0.39–1.35	Commercial	Enteric fermentation (39–60%)	Manure management (29–58%)	NA	LCA	Australia	36
1.34	Intensive	Feed production	Manure management	NA	LCA	China	37
1.12	Intensive	Raw milk production (75.27%)	Dairy processing (15.45%)	Packaging disposal (5.89)	LCA	China	38
1.11	Intensive	Enteric fermentation (57%)	Manure management (10%)	Concentrate feed production (8%)	LCA	Australia	39

LCA: life cycle assessment; NA: not available; NL: Netherland; NZ: New-Zealand; Syst. Rev: systematic review.

It is evident that CH_4_ emission intensity varies between production systems^27^^,^^29^^,^^40^^,^^41^ and per kg output (FPCM) per lactation^25^ and farm management practices.^24^^,^^30^ According to Gerber et al.,^42^ GHG emissions increase with higher yield, but emissions per kg FPCM decrease significantly as animal productivity increases. It is shown that the CF of milk production is more than 2-fold higher when milk yield is below 3500 kg a year per lactating cow.^25^ Depending on the production system, CF for milk has been estimated to be as low as 0.75 and as high as 1.21, and even over 5.0 kg of CO_2_e/kg energy corrected milk (ECM) or FPCM.^29^^,^^41^ Likewise, the CF of milk ranged from 0.74 (New Zealand) to 5.99 (Tanzania) kg CO2e per kg^−1^ FPCM.^29^ In the Peruvian Amazon, silvo-pastoral dairy production systems have an average milk CF of 2.26 kg CO_2_e/kg FPCM,^26^ while smallholder dairy farms in Ethiopia have a milk CF of 2.35 kg CO_2_e/kg FPCM.^27^ A more recent investigation revealed that housed-farms exhibited elevated CF compared to mixed and grazing farms, with farm management practices accounting for up to 79% of the variability in CF.^24^ In this context, increasing the length of the grazing season and improving annual milk production per hectare and per cow can reduce the CF of milk and increase farm profit.^43^

Nevertheless, promoting balanced feed rations and adjusting concentrate feeding depending on cows’ needs throughout the lactation cycle is suggested as an alternative strategy to increase milk production and reduce CF per milk output on smallholder farms.^32^ It is worth noting that farms with superior feed quality, higher production levels, and a greater percentage of lactating animals tended to have lower milk CF values (1.76 *vs.* 3.09 kg CO_2_e/kg FPCM).^26^ On the other hand, improving milking cow productivity and increasing the proportion of milking cows, as well as implementing various manure management systems and encouraging dairy farmers to return manure to nearby crop lands, were identified as promising measures to decrease environmental impacts.^37^ Therefore, farm systems that optimize milk production have the lowest environmental impact.^34^ It has been determined that the calculated emissions of CO_2_e per kg of FPCM produced vary between 0.8 and 3.5 kg CO_2_e/kg FPCM (Table 1). Different production systems and feeding strategies exert diverse impacts on the CF, where intensive systems exhibit elevated emissions compared to pasture-based or smallholder farms (Table 1). Summarized studies focusing on CF and milk production in dairy cattle have pinpointed enteric fermentation, manure management, and feed production as primary contributors to emissions (Table 1). Within this framework, the proportionate contribution of various emission sources varies between countries, with enteric fermentation emerging as a significant contributor in the majority of cases. Overall, these studies underscore the need for implementing sustainable practices in dairy cattle production to curtail carbon emissions and alleviate environmental repercussions.

## Genetic basis of methane emission traits in dairy cattle

Methane emission traits are partially influenced by genetics, with the surrounding environment being the most important factor.^10^ Genetic selection for traits related to CH_4_ emission has the potential to bring about permanent and cumulative changes. The literature (Table 2) highlights the presence of sizable genetic variation in CH_4_ emission traits among dairy cattle, offering valuable insight for inclusion in genetic selection programs.^12–14^^,^^44^^,^^52^ One way to evaluate the nature and extent of genetic variation for methane emissions is to genetically evaluate traits, such as CH_4_ intensity (MeI: methane per unit of product), CH_4_ yield (MeY: methane per unit of feed intake), CH_4_ production (MeP: the total amount of CH_4_ emitted by cows in grams per day), and residual methane traits corrected for dry matter intake and milk yield in dairy cattle.

**Table 2.** Summary of recent studies on estimates of heritability for methane emission traits in dairy cattle.

Trait	Breed	Number of animals	Heritability (*h*^2^ ± *SE*)	References
MeY	HF	287	0.27 ± 0.12	17
	NA	NA	0.24 ± 0.04	44
MeP	HF	451	0.36 ± 0.12	45
	HF	330	0.16 ± 0.10	17
	HF	575	0.12 ± 0.03	19
	NA	NA	0.21 ± 0.01	44
	HF	1501	0.12 ± 0.04	46
MeC	HF	4664	0.17 ± 0.04	47
	HF	647	0.15 ± 0.03	19
	HF	1501	0.11 ± 0.03	46
	HF	337	0.12 ± 0.01	48
MeIc	HF	1956	0.04 ± 0.03	19
	NA	NA	0.18 ± 0.01	44
MeIp	HF	1839	0.04 ± 0.03	19
MeI	HF	265	0.21 ± 0.14	17
PME	Walloon dairy cows	229,465	0.14 ± 0.05*	49
LMI	Walloon dairy cows	229,465	0.24 ± 0.05*	49
CH_4_/CO_2_	HF	182	0.12 ± 0.14	50
RT	HF	363	0.45 ± 0.14	45
	HF	1486	0.14 ± 0.03	51
	HF	1501	0.17 ± 0.06	46

MeY: methane yield; MeP: methane production; MeC: methane concentration; MeIc: methane intensity calculated using MeC; MeIp: methane intensity calculated using MeP; MeI: methane intensity; PME: predicted daily methane emission; LMI: log-transformed predicted CH_4_ intensity; RT: rumination time; HF: Holstein Friesian. NA stands that the study did not specify the breed used in the meta-analysis, nor did it provide specific breed information and number of animals involved.

*Indicates that values are in standard deviation.

In an attempt to perform a meta-analysis to estimate genetic parameters for methane emission traits (MeY, MeI, and MeP), Hossein-Zadeh^44^ confirmed the presence of modest genetic variation for methane emission traits with heritability estimates ranging from 0.180 to 0.244 in dairy cows. Recently, Richardson et al.^53^ reported heritability estimates of 0.16 for MeP, 0.23 for MeY, and 0.33 for MeI in Australian Holstein cows. Similarly, Lassen and Løvendahl^13^ reported a heritability of 0.21 for both CH_4_ production and CH_4_ intensity recorded over a week in Danish Holstein cows from multiple commercial herds. However, Pszczola et al.^15^ reported higher heritability estimates for daily MeP with values ranging from 0.23 to 0.30 across lactation in two herds. Using longitudinal data, Difford et al.^54^ reported heritability estimates that varied from low (0.15) to moderate (0.26) for MeC in Dutch and Danish Holstein cows, respectively. In contrast, Manzanilla-Pech et al.^19^ reported heritability estimates of 0.14 for MeC and 0.15 for MeP in Danish Holstein population. Furthermore, heritability estimates of 0.12 were reported for CH_4_/CO_2_^50^ and methane concentration^48^ in dairy cattle. For various definitions of residual methane, Richardson et al.^53^ reported heritability estimates ranging from 0.11 to 0.21 in Australian Holstein cattle. Considering predicted methane yields as CH_4_ traits, heritability estimates varied from 0.12 to 0.44 in Dutch Holstein-Friesian cows, thus, indicating that response to selection would be expected to reduce predicted CH_4_ emission (PME).^55^ Kamalanathan et al.^17^ reported heritability estimates of 0.16 for daily MeP, 0.27 for daily MeY, and 0.21 for daily MeI in dairy cattle, indicating that there is room to reduce CH_4_ emission in Holstein cattle through genetic selection.

On the other hand, there is evidence of genetic control of microbiota composition through changes in physical and physiological conditions in the rumen that promote the growth of specific microbes.^13^^,^^56^ It is of note that, the specific functional capacity of the ruminal microbiome is heritable with heritability estimates ranging between 0.13 and 0.61 for the comprehensive set of microbial genes involved in a variety of CH_4_ metabolism pathways and ribosomal biosynthesis.^57^ Likewise, heritability estimates for the relative abundance of rumen microbes ranged between 0.08 and 0.48,^48^ which opens up opportunities to consider a new source of genetic variation that could be utilized in the dairy cattle genetic selection program. This suggests that gaining insights into the genetic basis of the microbiota and its composition with CH_4_ emissions holds great importance.

When considering genetic correlations, it is important to note that traits like milk production, composition, body weight, and health status of cows are genetically linked to CH_4_ production.^7^ Previous studies have shown unfavorable associations between CH_4_ production and milk production,^13^^,^^44^^,^^54^^,^^58^ dry matter intake,^54^^,^^58^ and residual feed intake^12^ in dairy cattle. Therefore, caution should be advised when selecting for reduced CH_4_ production to avoid a decrease in milk yield. Several other studies have examined correlations between CH_4_ emissions and other breeding goal traits, and have found that selecting for reduced CH_4_ emissions is likely to have minimal consequences on other traits, such as reproduction and health.^7^^,^^59^

## Diet, rumen microbial community, and emissions from enteric methane in dairy cattle

**Figure 1.**
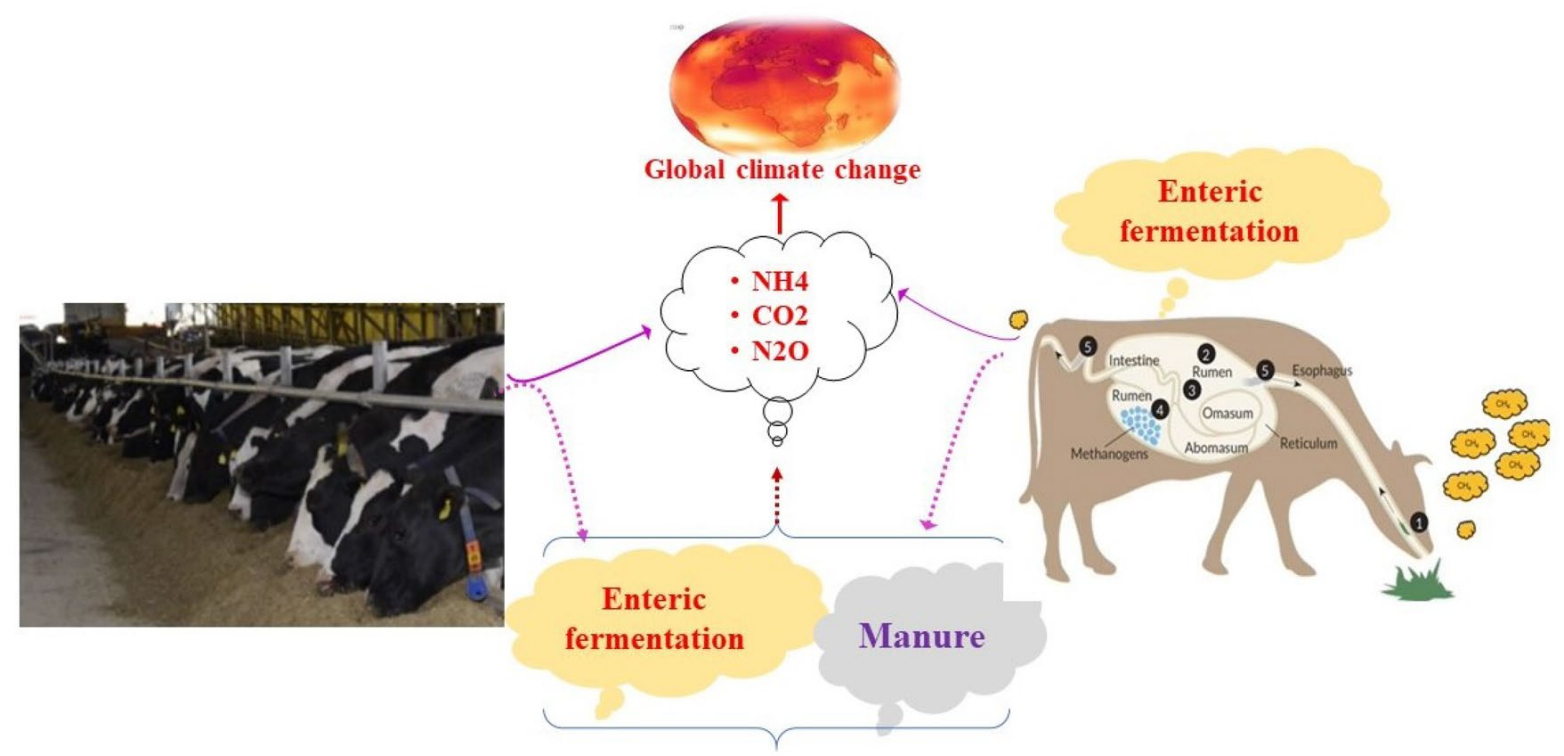
An overview of animal feed, rumen microbiota and enteric methane emissions in dairy cattle.

It is widely acknowledged that diet plays a significant role in shaping the composition of the rumen microbial community.^60–62^ In ruminants, the rumen, one of their stomachs, stands out for harboring a diverse community of bacteria, ciliate protozoa, and anaerobic fungi that work together to break down feed material (Fig. 1). In this process, complex dietary carbohydrates, proteins, and lipids are converted into volatile fatty acids (VFAs), microbial proteins and vitamins, while CO_2_, H_2_, and other compounds are released.^57^^,^^63^ Among the VFAs, acetate and butyrate are produced through methanogenic fermentation, which spares H_2_, while propionate is glucogenic, utilizes H_2_ during enteric fermentation.^64–68^ This indicates that increased acetate and butyrate production during enteric fermentation leads to higher production of H_2_ and CO_2_ by methanogenic bacteria that cannot be utilized by dairy cows, as a result, CH_4_ is generated as a by-product, which the cow expels through eructation.^69^ Eructated CH_4_ not only significantly contributes to global warming, but also represents a 5–7% loss of dietary energy.^70^ It is estimated to account for between 2% and 12% of net energy intake, which has a profound impact on dairy cattle productivity^12^. As suggested by Gerber et al.,^42^ when low-quality feed spends a long time in the rumen, it leads to high enteric methane emission and emission intensity, resulting in low animal productivity. For instance, the inclusion of a high percentage of forage in the ration (58.6%) explains the relatively increased emissions from enteric fermentation.^23^ Therefore, changes in feeding practices, such as the use of concentrates and high-quality fodders like legumes and high-starch forages, have been associated with reduced CH_4_ emission in ruminant digestive systems.^71^^,^^72^ Indeed, the intricate relationship between rumen microbiota, their functional composition and H_2_ is further influenced by various factors, such as diet, host genetics, physiological status, dietary composition, and environmental conditions.^61^^,^^73^ These factors are crucial in determining animal performance and efficiency.^74^

Implementing balanced feeding strategies to reduce CH_4_ emissions from dairy cows is of great importance from both an environmental and economic perspective. One way to achieve this is improving the efficiency of dairy cattle in converting feed to milk, rather than wasting energy as enteric methane.^11^ It is important to note that, altering the diets fed to ruminants leads to changes in CH_4_ emissions and alterations in the VFA proportions.^75^ In this context, modulating the rumen microbiome to improve feed efficiency and reduce enteric CH_4_ emissions from ruminants in general and dairy cattle in particular, without altering other traits, such as production and health is desirable as a strategy to decarbonize the dairy industry and improve its productive efficiency.

Rumen metabolites, such as VFAs indicators, have the potential to be used as proxies (indirect indicators/traits) for CH_4_ emissions, mostly because the fermentation of feed in the rumen into VFAs is connected to the release of H_2_.^76^ Diets that promote propionate dominated fermentation in the rumen have been linked to decreased CH_4_ emissions, while diets that favor acetate/butyrate dominated fermentation are associated with increased CH_4_ production.^77^ On the other hand, supplementing ruminants with dietary lipids can act as a toxic agent against methanogens and protozoa, leading to a change in the rumen environment, resulting in increased propionate production, and reduced enteric CH_4_ emissions.^71^ Adding lipids to the diet can reduce absolute CH_4_ emissions by 19%.^3^ It is important to note that diet composition and dry matter intake are key factors that explain the variation in CH_4_ emissions. Among the main VFAs, propionate is generally associated with diets that have lower CH_4_ emissions, while butyrate and acetate are correlated with diets that have higher CH_4_ yield.^67^ CH_4_ emissions are also generally related to the ratio of acetate to propionate (A:P), with decreasing CH_4_ production associated with a decreasing A:P ratio.^67^ Numerous studies have also shown that lowering the dietary CP content resulted in a significant reduction in N_2_ excretion of dairy cattle, which subsequently lowers ammonia (NH_3_) and nitrous oxide (N_2_O) emissions related to energy-corrected milk and DM intake.^78–81^ Therefore, comprehensive feeding strategies, such as begin-of-pipe feeding mitigation measures must be developed and implemented to mitigate the harmful effects of GHG emissions.^81^ In this regard, efforts are underway to develop and implement the possibilities of CP-optimized feeding strategies for dairy cows.^82^ While Alfalfa is widely recognized as the predominantly used forage crop for dairy cattle and is considered a substantial carbon sink owing to its high productivity, extensive root systems, and nitrogen-fixation capacity, the emissions N_2_O can notably diminish its potential in mitigating climate change.^83^

## Methane-reducing cow feed additives

Several novel and interesting feed additives for cattle have been suggested to reduce enteric methane emissions. For instance, methane inhibitors, such as monensin, 3-Nitrooxypropanol (3-NOP), and bromoform-containing seaweed species have shown promise in reducing emissions in cattle.

Evidences shows that 3-NOP effectively reduces the formation of enteric CH_4_ in dairy cows by inhibiting the enzyme methyl-coenzyme M reductase.^73^ Studies have demonstrated that supplementing high yielding dairy cows with 3-NOP, a powerful CH_4_ inhibitor, can lead to a significant decrease of 23–37% in CH_4_ emissions.^84^^,^^85^ Supporting these findings, Melgar et al.^86^ have consistently shown that administering 3-NOP through the total mixed ration (TMR) can reduce CH_4_ emissions by 22–40% in lactating dairy cows. This reduction is attributed to the high solubility and rapid metabolism of 3-NOP in the rumen, resulting in decreased enteric MeP. Importantly, the use of 3-NOP reduce CH_4_ emissions in cows does not have a negative impact on animal feed intake or productivity.^71^^,^^87^^,^^88^ However, it does show a linear relationship with an increase in both milk fat concentration and yield.^86^ In line with this, a meta-analysis conducted at Pennsylvania State University, which included studies involving dairy cows fed the CH_4_ inhibitor 3-NOP, consistently demonstrated a 28–32% reduction in daily CH_4_ emissions yield and intensity.^89^ This reduction was observed without any effect on DMI, milk production, and body weight, but there was a slight increase in milk fat concentration (0.19 percentage units) and yield.^89^ A recent meta-analysis of models by Kebreab et al.^90^ observed that a 3-NOP dose of 70.5 mg/kg DM resulted in decreases in MeP (g/day), MeY (g/kg DMI), and MeI (g/kg energy-corrected milk) of 32.7, 30.9, and 32.6%, respectively. Moreover, evidence suggests that early-life dietary intervention in dairy calves, such as supplementing their diets with 3-NOP, can lead to a long-term reduction in CH_4_ emissions. This indicates that changes in microbial colonization of the rumen before weaning may imprint the rumen microbiome and have lasting effects on phenotypes later in life.^91^ According to Vrancken et al.,^92^ several companies have started manufacturing commercial feed additives of this kind, which may become available in the market in the next few years.

Regarding of the use of macroalgae, the incorporation of *Asparagopsis taxiformis* (AT) into the diets of dairy cows and steers can significantly reduce enteric MeP by 9–98%.^71^^,^^93^ In another study by Ramin et al.,^94^ AT supplementation in dairy cow diets resulted in a 61% reduction in enteric MeP. However, the widespread use of seaweed feed additives to mitigate livestock CH_4_ emissions poses certain concerns. One argument suggests that to have a substantial global impact, there would need to be massive production and sale of seaweed supplements, considering the large worldwide population of dairy cattle. Harvesting enough seaweed for incorporation into their feed would therefore be logistically impossible. To use seaweed as a feed additive on a large scale, cultivation in aquaculture operations would thus be necessary.^40^ Another challenge is the palatability of seaweed to cows, as researchers have found that cows do not enjoy the taste of seaweed. Additionally, concerns have been raised about the long-term effects of seaweed, such as the presence of bromoform in *Asparagopsis taxiformis* which can be excreted in milk and urine, and potentially cause inflammation in the rumen wall. These concerns raise questions about the safety of seaweed for humans and the environment.^3^^,^^95^ In a nutshell, these additives are now undergoing testing for application in dairy cows due to their considerable potential. For instance, companies like CH_4_ Australia are commercially introducing *Asparagopsis* seaweed to address climate change.

On the other hand, when dairy cows were supplemented with garlic and citrus extract, rumen fermentation and microbiota, were altered, leading to decreased MeP and MeI without affecting dry matter intake or milk production in dairy cows.^96^

## Genomic regions and candidate genes associated with methane emission traits

Understanding the genetic basis of methane emission traits is crucial for implementing sustainable breeding strategies aimed at reducing GHG emissions from livestock. Quantitative traits, including CH_4_ emission traits in farm animals, are intricately linked to polygenes located at QTLs, distributed widely throughout the genome. Within these QTLs, pinpointing a handful of markers that adequately explain a substantial proportion of genetic variance poses a considerable challenge. Consequently, the candidate gene approach has proven notably unsuccessful in the realm of commercial breeding for quantitative traits. Despite the fact that CH_4_ emissions are a complex trait influenced by multiple genetic loci acting together, only a limited number of QTLs associated with CH_4_ emissions are currently listed on the QTLdb website, primarily due to the limited routine measurement of CH_4_ emissions in dairy cattle.^11^ This underscores the pressing need for comprehensive identification of genome-wide loci and/or variants that are associated with the complex phenotypes of CH_4_ emissions through the use of GWAS and transcription wide association studies (TWAS).

Given recent developments in high-throughput sequencing techniques and availability of high-resolution sequence data, it is now feasible to detect numerous significant genomic variants that impact CH_4_-emission traits in dairy cattle. However, the identification of genome-wide SNPs and genomic regions associated with CH_4_ emission traits in dairy cattle through GWAS is still in its early stages. Nevertheless, it is gaining momentum due to the increasing challenges posed by climate change, as evidenced by the recent surge in published studies (Table 3).^7^^,^^11^^,^^18^^,^^19^^,^^49^ For example, Atashi et al.^49^ identified several genes, namely *LY6D*, *GML*, *CYHR1*, *PPP1R16A*, *ARHGAP39*, *ZNF7*, *OPLAH*, *MAF1*, and *SPATC1* located on chromosome 14, as potential candidate genes associated with predicted daily CH_4_ emissions and log-transformed predicted MeI. Similarly, Pszczola et al.^7^ reported potential QTL regions and their respective candidate genes *CYP51A1* (BTA4: 9,306,414–9,323,252), *PPP1R16B* (BTA13: 68,258,627–68,366,080), *NTHL1* (BTA25: 1,590,252–1,595,934), *TSC2* (BTA25: 1,596,730–1,626,967), and *PKD1* (BTA 25: 1,627,978–1,666,088) affecting CH_4_ production located within QTLs that are related to feed efficiency, milk-related traits, body size and health status in dairy cattle. Importantly, *PKD1* was identified as one of the most promising genes associated with digestive development and potentially involved in GHG emissions, including not only CH_4_ but also nitrogen-related emissions.^7^ Manzanilla-Pech et al.^19^ reported the presence of strong association signals on BTA13 and 26 for MeC and MeP in Danish Holstein cattle. Several previous studies have also confirmed significant effects of QTL regions on BTA1, 3, 13, and 20 that are strongly associated with MeP in dairy crossbred-dual purpose Mexican cows. Furthermore, Manzanilla-Pech et al.^19^ reported significant regions on chromosomes 3, 6, and 13 for MeP, MeY, and MeI in Australian Holstein cows. Van Engelen^55^ reported strong associations for MeP and MeY with the SNPs identified as the ones coding for *DGAT1* on chromosome 14. There appears to be stronger evidence, suggesting a reliable association on chromosome 13 for MeP in several populations of Holstein cows in different countries.^7^^,^^18^^,^^19^ On the other hand, Sarghale et al.^11^ identified the most significant (*p* < 5 × 10^−8^) SNPs and regions associated with predicted CH_4_ emission per kg milk (15: 25,797,132) and per kg fat (19: 24,494,923 and 28: 21,771,233) in dairy cattle that are in close proximity to the QTLs. Additionally, promising candidate genes (*ARPC1A* and *TRRAP*) related to residual feed intake^99^^,^^100^ were identified for valeric acid.^11^ The *ARPC1A* gene is involved in regulating branched actin networks, whereas *TRRAP* is an adaptor protein found in multiprotein chromatin complexes with histone acetyltransferase activity, which adds a specific tag for epigenetic transcription activation.^11^

**Table 3.** Candidate genes and SNPs in significant regions associated with various methane emission traits in dairy cattle.

Ch	Candidate genes/SNPs	Position (bp)	Type of study	Traits associated with	Breed	Number of genotyped animals	Country	References
1	ARS-BFGL-NGS-93180	138,832,098	GWAS	RMETc, MeY	HF	1962	Denmark	19
4	4: 115,131,249	115,131,249	GWAS	PME	HF	150	Iran	11
	ARS-BFGL-NGS-24888	3,583,133	GWAS	MeC, MeP	HF	1962	Denmark	19
	Hapmap59221-rs29014908	35,939,871		MeIc, MeIp				
	Hapmap44201-BTA-114510	36,842,170		MeIc, MeIp				
	*CYP51A1*	9,306,414–9,323,252	GWAS	MeP	HF	287	Poland	7
5	5: 16,795,260	16,795,260	GWAS	Valeric acid	HF	150	Iran	11
6	Hapmap51046-BTA-75812	61,984,747	GWAS	MeC, MeP	HF	1962	Denmark	19
	Hapmap52436-rs29009653	99,732,094		MeIc, MeIp				
11	ARS-BFGL-NGS-47330	44,562,022	GWAS	MeC, MeP	HF	1962	Denmark	19
	ARS-BFGL-NGS-12929	64,313,748		MeC, MeP				
	Hapmap26463-BTA-159947	92,086,008		MeC, MeP				
13	13: 81,673,732	81,673,732	GWAS	PME	HF	150	Iran	11
	Hapmap49571-BTA-32781	47,583,553	GWAS	MeC, MeP	HF	1962	Denmark	19
	BTB-00525367	47,915,618		MeC, MeP				
	ARS-BFGL-NGS-70206	48,622,655		MeC, MeP				
	BTA-115847-no-rs	48,826,815		MeC, MeP				
	*PPP1R16B*	68,258,627–68,366,080	GWAS	MeP	HF	287	Poland	7
	*SLC23A2*	47,642,260	GWAS	MeP	*Bos taurus*, *Bos indicus*, and crosses	280	Mexico	18
14	14: 62,204,044	62,204,044	GWAS	MeP	*Bos taurus*, *Bos indicus*, and crosses	280	Mexico	18
	*LY6D, GML, CYHR1, PPP1R16A, ARHGAP39, ZNF7, OPLAH, MAF1, and SPATC1*	1.86–2.12 Mb 1.48–1.68 Mb	GWAS	PME LMI	Walloon-dairy cows	7381	Belgium	49
	*TRPS1*	48,890,778, 48,858,864, 48,726,666, 48,679,326	GWAS	CH_4_ ppm /d	HF	483	Poland	97
15	15: 25,797,132	25,797,132	GWAS	PME	HF	150	Iran	11
	BTA-37116-no-rs	57,228,610	GWAS	MeC, MeP	HF	1962	Denmark	19
18	ARS-BFGL-NGS-32691	34,159,637	GWAS	MeC, MeP	HF	1962	Denmark	19
	ARS-BFGL-NGS-54767	7,605,307		MeIc, MeIp				
19	UA-IFASA-7562	49,438,164	GWAS	MeC, MeP	HF	1962	Denmark	19
	19: 24,494,923	24,494,923	GWAS	PME	HF	150	Iran	11
	*RAI14*	47,747,001	GWAS	MeP	*Bos taurus*, *Bos indicus*, and crosses	280	Mexico	18
24	ARS-BFGL-NGS-103202	61,455,723	GWAS	MeYc, MeYp	HF	1962	Denmark	19
25	*NTHL1*	1,590,252–1,595,934	GWAS	MeP	HF	287	Poland	7
	*TSC2*	1,596,730–1,626,967		MeP				
	*PKD1*	1,627,978–1,666,088		MeP				
	25: 37,967,076	37,967,076	GWAS	Valeric acid	HF	150	Iran	11
26	Hapmap33073-BTA-162864	21,180,893	GWAS	MeC, MeP	HF	1962	Denmark	19
	ARS-BFGL-NGS-2180	24,477,962		MeC, MeP				
	ARS-BFGL-NGS-1092	24,531,763		MeC, MeP				
	ARS-BFGL-NGS-18194	24,575,207		MeC, MeP				
	ARS-BFGL-NGS-81009	26,491,674		MeC, MeP				
	Hapmap38478-BTA-20824	28,723,721		MeC, MeP				
	Hapmap40449-BTA-61103	31,213,256		MeC, MeP				
27	Hapmap19519-rs29022379	19,017,466	GWAS	MeIp, MeYc	HF	1962	Denmark	19
28	28: 21,771,233	21,771,233	GWAS	PME	HF	150	Iran	11
	ARS-BFGL-NGS-60192	25,609,489	GWAS	MeC, MeP	HF	1962	Denmark	19
29	ARS-BFGL-NGS-24205	25,325,889	GWAS	MeC, MeP	HF	1962	Denmark	19
	*ABS4* and *DNAJC10*	–	Metha-genome	Rumen microbiota composition	HF	690	Denmark	98

Ch: chromosome; RMETc: residual methane based on methane concentration (MeC); MeY: methane yield; PME: predicted daily methane emissions; MeC: methane concentration; MeP: methane production; MeIc: methane intensity based on MeC; MeIp: methane intensity based on MeP; LMI: log-transformed predicted CH_4_ intensity.

Most GWAS studies on CH_4_ traits in dairy cattle have generally had small number of genotyped animals, with sample sizes ranging from fewer than 300 cows to <2000 cows. This is far below the optimal power for detecting QTL explaining at least 5% of the genetic variance, so care should be taken when considering for reported candidate variants. According to Gebreyesus et al.^101^ who performed a power detection test on several Holstein populations, as a function of sample size and proportion of explained variance by a QTL, a population of 2,880 animals could have a detection power of 0.97, whereas a population of 1566 animals could have a detection power of 0.57 and a population of 614 animals explaining 5% of the genetic variance. This indicates more CH_4_ data are required to achieve higher power detection in GWAS for QTL associated with CH_4_ traits with the corresponding genetic variance associated with it.

Several studies have emphasized the importance of ruminal VFAs as metabolic biomarkers for CH_4_ emissions in dairy cattle. Interestingly, most of the SNPs that have a strong association with valeric acid (BTA5, 11, and 25) and isovaleric acid (BTA9 and 28) in dairy cattle are found near QTLs for milk production, its components, body weight, and RFI.^11^ These metabolic biomarkers, such as valeric acid and isovaleric acid, can assist dairy farmers in improving the availability of these acids in the rumen for ruminal cellulolytic bacteria through genetic selection.

There is increasing evidence that cow host genetics play a role in shaping the composition of the rumen microbiota.^5^^,^^102^ Understanding how host genetics influence variation in the ruminal microbiota and their combined effects on CH_4_ emissions is crucial for breeding dairy cows that produce less CH_4_ .^5^^,^^48^^,^^57^^,^^66^^,^^98^^,^^102^ The ruminal bacterial community is believed to be influenced by a large number of genes, making it a polygenic trait. However, the complex relationship between cow genes and microbial relative abundance in the rumen is not yet well documented. Transcriptome-wide and GWAS can be used to pinpoint host chromosomal regions and causal genes that affect the composition and function of the rumen microbiome. Recent advancements in high-throughput sequencing techniques and advanced analytical methods have provided opportunities to unravel the rumen biochemical network involved in CH_4_ production in cattle. In a recent GWAS conducted by Abbas et al.,^103^ several loci on different chromosomes (2, 6, 9, 19, 23, and 27) were found to be associated with the rumen microbiota. Notably, *Prevotella*, a dominant genus in the rumen that is involved in protein and energy metabolism, showed associations with these loci. The study also revealed a connection between the *CLDN18* gene on chromosome 1, within the 132–133 Mb region, and *Succiniclasticum ruminis*, a common rumen inhabitant, that specializes in converting *succinate* to *propionate* for primary energy production. Abbas et al.^103^ also highlighted specific genetic associations between the genes *GMDS* (chromosome 23), *A4GNT* (chromosome 1), and *ANXA5* (chromosome 6), and *Succiniclasticum ruminis* and *Prevotella*. These genes are involved in endocytotic and exocytotic pathways, suggesting their role in modulating host rumen microbes to enhance energy absorption. The candidate genes *DNAH9*, *ABS4*, and *DNAJC10* have been identified as potentially influencing and modifying the composition of ruminal microbial communities, making them potential genetic markers in cows.^98^ The abundance of *TSTA3* was host-genomically found to be positively correlated with CH_4_ emissions and could be a valuable biomarker for rumen pH, CH_4_ metabolism, and potentially for host-microbiome interactions to enhance saliva production.^57^

Notably, understanding the specific molecular and biological pathways that regulate CH_4_ production is crucial for investigating the mechanisms of reduced CH_4_ emissions in dairy cattle. These pathways may be connected to energy metabolism, feed efficiency, and nutrient utilization, which are important factors to consider. To develop effective genetic selection strategies that address both CH_4_ emissions and dairy cattle productivity, it is essential to have a comprehensive understanding of these pathways. Candidate genes identified through GWAS are often associated with specific molecular pathways and biological functions, which further emphasizes the importance of studying them.

## Breeding strategies to reduce CH_4_ emissions in dairy cattle

**Figure 2.**
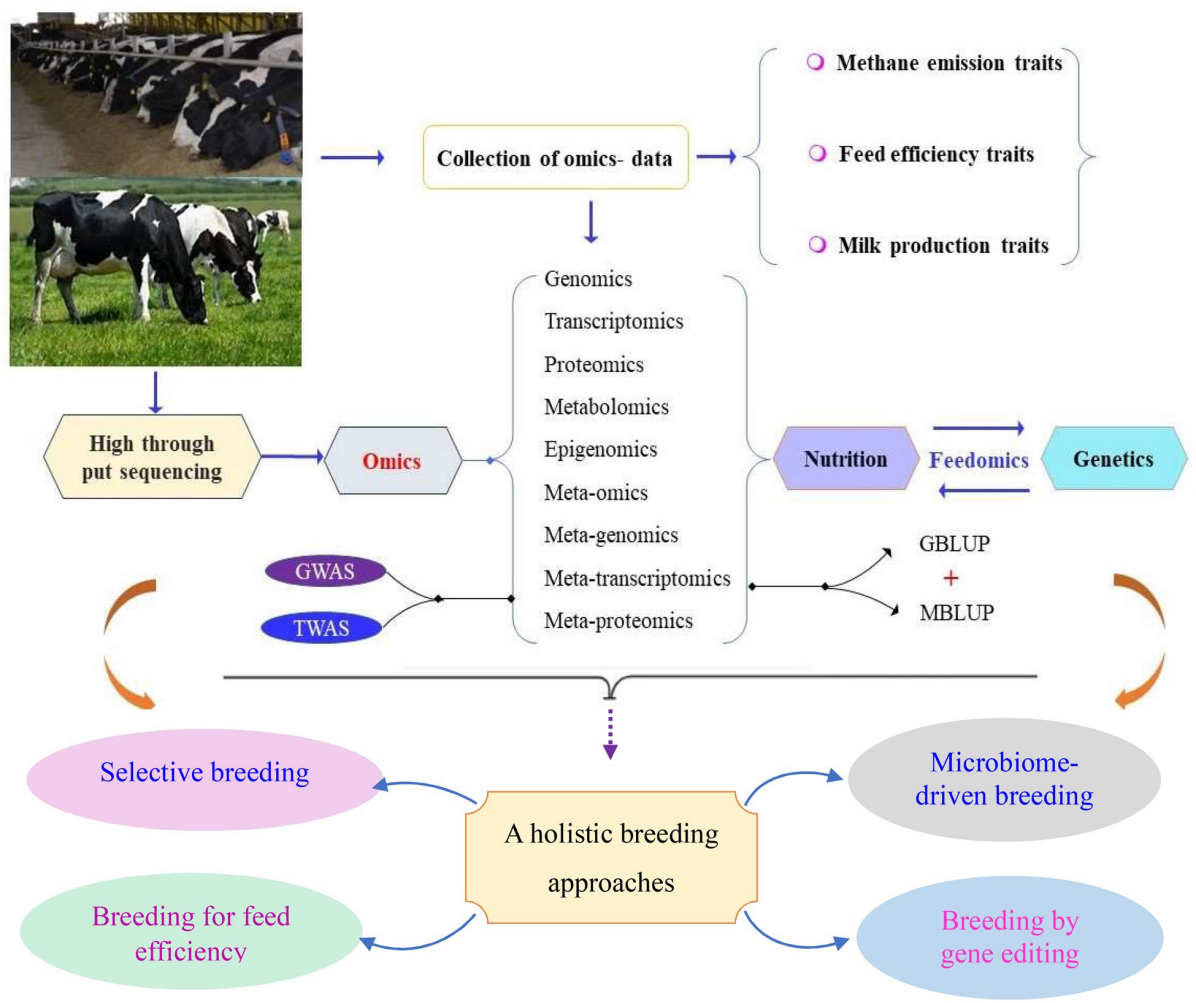
Brief overview of integrating omics data collection and selection with genomic best linear unbased prediction (GBLUP) and microbiome best linear unbiased prediction (MBLUP) methods, and holistic breeding strategies to achieve optimal genetic progress for low methane emission in dairy cattle.

The future production environment is anticipated to undergo changes as the modern dairy production system heavily relies on expensive inputs, such as grain. Factors, such as ongoing competition for grain for human consumption, particularly in most agriculture dependent countries, and the impact of climate change on grain production are expected to drive grain prices higher. These economic considerations may lead to alterations in dairy production systems and subsequently influence the desired genetic attributes. Given the unpredictability of the future, genetic improvement systems that can swiftly adapt to changes in breeding objectives are deemed necessary.^104^ A more enduring solution might come through gene editing combined with selective breeding, which could provide a long-term solution for methane emissions.^1^ A more reasonable attention should be placed on a holistic breeding approach, including incorporating feed conversion efficiency and methane emission traits into the breeding goal traits (Fig. 2). This approach should be implemented to confront future emerging challenges, and ensure sustainable dairy production.

### Selective breeding

Genetic selection stands out as a viable approach to address CH_4_ emissions, given the considerable genetic variation observed in both CH_4_ emissions and production (g/day) traits in cattle. The heritability of CH_4_ exhibits substantial genetic variation, with values ranging from 0.12 to 0.45.^12–14^^,^^19^ The strategy to reduce CH_4_ production involves selection for CH_4_ traits, either through direct CH_4_ measurements or by breeding based on correlated traits, such as residual feed intake.^93^ Although breeding for increased milk production has already contributed to a reduction in emission intensity, more rapid decreases in emission levels could be achieved by selecting traits more strongly correlated with CH_4_ emissions than with production.^104^ A compelling recent investigation by Zira et al.^105^ revealed that when cows are selected based on resilience traits, the CF per unit of milk and meat product is reduced compared to those selected under the current breeding goal, particularly in situations of constrained feeding conditions. Their conclusion emphasizes the significance of development of breeding goals that address resilience to disturbances arising from climate changes as a crucial aspect of promoting sustainable dairy production. However, CH_4_ emission traits do not take into account in the current dairy breeding goal traits in dairy cattle. If this trend persists, CH_4_ production is projected to increase by 13% by 2050.^3^ Therefore, the inclusion of CH_4_ traits into national breeding programs, alongside other breeding goal, holds the potential to reduce CH_4_ production. However, a tradeoff may exist, potentially leading to a decline in the genetic trend for milk production^9^ and reduced fertility and body condition score,^106^ necessitate more profound adjustments in current indexes.^107^ A practical example comes from Spanish Holstein cattle, where a feasible 20% reduction in CH_4_ emissions over a decade was achieved, with a corresponding reduction in gain for production traits ranging from 6% to 18%.^108^ Consequently, the inclusion of CH_4_ emission traits in commercial selection indices may have consequences on other economically important traits in dairy cattle. When constructing these indices, careful consideration of the implications of GHG emissions is essential, as different sub-indexes can have varying effects on emissions.^109^ Nevertheless, additional research is required to assess the downstream and upstream net GHG emissions impacts of selecting based on CH_4_ measurements.^93^ Likewise, further investigation is needed to understand the relationship between CH_4_ emissions, feed intake, and production traits.

Breeding for reduced CH_4_ production offers a long-term solution that will define the breeds pursued for this approach indefinitely. However, the pursuit of lower CH_4_ emissions is not governed by a single gene; rather, the phenotype could be conferred by the interaction of several genes, each exerting a small effect. The elucidation of the genetic architecture of CH_4_ emission traits is now possible through genomics and provides a robust tool to address these challenges. In this regard, genomic selection is often preferred due to its ability to capture the polygenic nature of these traits. Indeed, the prospects of developing dairy cattle with minimal CH_4_ emission are possible through genomic selection. Genomic selection, employing genome-wide DNA markers, holds an advantage over conventional selection methods, particularly for selecting animals early in life and for traits that are challenging or expensive to measure, such as CH_4_ emissions and feed conversion efficiency in dairy cattle. To date, there is a lack of reports on genomic selection targeting lower CH_4_ emissions while concurrently maintaining production levels in dairy cattle.

### Microbiome-driven breeding

Apart from studying traits associated with genome-wide genetic variations, it is crucial to acknowledge that livestock harbor various symbiotic organisms, including bacteria and protozoa, in their digestive tract.^104^ Profiling the rumen microbiome has historically been challenging due to difficulties in culturing many rumen bacterial species. However, advances in high-throughput-sequencing technology and improvements in analytical capabilities are now facilitating the profiling of the rumen microbiome in animals. It is worth mentioning that researchers are currently investigating QTL in hosts that influence the composition of the gut microbiome in dairy cattle. Consequently, the selection of dairy cattle could involve selecting animals with a desirable rumen microbial ecosystem, and directly influencing these microbes could improve host efficiency.

The literature suggests that the abundance of microbial communities, including their genes and interactions, serves as effective biomarkers for predicting phenotypes associated with CH_4_ emissions.^57^ In addition, there is evidence indicating genetic control over the composition of microbiota in the rumen, influenced by changes in both physical and physiological conditions that favor the growth of specific microbes.^13^^,^^56^^,^^103^ In this context, Martínez-Álvaro et al.^57^ have provided evidence of how genomic selection can modify the communities and functions of the rumen microbiome to develop animals with lower CH_4_ emissions. Their findings highlighted that specific microbial gene abundances offer more valuable information for breeding purposes compared to specific taxonomies. This observation is supported by the notion that a higher number of microbial genes, rather than genera, exhibit host-genomic correlations with CH_4_ emissions. By employing genome-resolved metagenomics, Roehe et al.^110^ identified 20 microbial gene abundances primarily linked to the methanogenesis pathway, elucidating 81% of the variance in CH_4_ emissions in cattle.^57^ The authors emphasized that selection based on the abundances of 30 microbial genes demonstrated a mitigation potential of 17% in mean CH_4_ emissions per generation. This mitigation potential surpassed the impact of direct genomic selection based on precisely measured CH_4_ emissions using respiration chambers (13%), highlights the potential effectiveness of a microbiome-driven breeding strategy in collectively reducing CH_4_ emissions and contributing to climate change mitigation.^57^

With recent omics-based advancements, regulatory ncRNAs expression has been studied in cattle, and it was found that miRNA expression shows site-specificity along the gut in cattle.^111^^,^^112^ Most tissue-specific miRNAs for ruminants can be found in the rumen.^111^ A study on early rumen development in neonatal dairy calves revealed that almost half of the expressed miRNAs are responsive to microbial metabolites,^113^ indicating their significant role as responders to the intestinal microbiome during early life stages. Manipulating early-life microbiomes may offer opportunities for long-term interventions aimed at improving cow CH_4_ emissions; however, there is still limited understanding of the regulatory mechanisms behind these crucial host-microbiome interactions during early-life stage. In summary, numerous factors including genetics, diet, age, and time of day contribute toward variations observed within rumen microbiomes among dairy cattle populations, which presents challenges for applying microbiome driven breeding strategies in this context.

### Breeding for feed efficiency

There is growing evidence supporting the idea that modifying the diet of dairy cows can effectively lower CH_4_ emissions. Pryce and Haile-Mariam^2^ have suggested that direct selection for feed efficiency presents an opportunity for rapid genetic progress in emission reduction. Notably, feed conversion efficiency confirmed a negative correlation with CF in dairy cattle.^32^ De Haas et al.^12^ proposed that by selecting more efficient cows, a 26% reduction could be achieved over a 10-year period. It has been observed that more efficient cows can produce milk without an increase in the amount of feed consumed. An international team of scientists conducted a recent meta-analysis of global data^87^ and proposed various product-based mitigation strategies. These include improving forage quality, specifically improving digestibility, and incorporating more concentrate feeds into the diet. These measures decreased enteric methane emissions in ruminants by 12–32%.

### Breeding by genome-editing

Genome editing is a powerful tool in genomics that has the potential to revolutionize animal production. In cattle, the main cause of CH_4_ emissions is the symbiotic microbes present in their digestive tract, rather than the cattle themselves. By directly manipulating the genome of methanogenic archaea, it is possible to engineer these microbes and explore new ways to reduce CH_4_ emissions from their rumen. It is possible to identify the specific genes involved in MeP in the rumen of cattle, it becomes easier to use gene editing techniques to either eliminate the bacteria that contribute high MeP or increase the expression of those that promote low-methane species,^1^ while maintaining proper digestion. This allows researchers to target specific genes involved in CH_4_ production and generate mutant methanogenic archaea that produce less CH_4_, which can then be used to populate the rumen of cattle. Previous research has demonstrated the feasibility of deleting the genomic sequence of *Methanosarcina acetivorans* and introducing multiple deletions causing Cas9-mediated genome editing.^114^ The genome sequence of Methanobrevibacter *ruminantium* is also available,^115^ and the functions of some predicted gene clusters have been established.^116^ Further research is needed on rumen methanogens to gain insights into the genes that contribute to MeP in the rumen. There are various actions that can expedite the implementation of gene-edited solutions for climate benefits, although the extent of their contribution is not yet clear.^1^ Innovative projects, such as the $70-million TED’s Audacious Project in California, aim to use CRISPR genome-editing on gut microbes in cattle to reduce emissions and halt climate change. These ground breaking initiatives demonstrate the potential for impactful research that will have a positive global impact. Addressing climate change through CRISPR genome editing and metagenomics research on microbiomes is crucial, as even the smallest organisms can provide valuable solutions to major challenges. Although the research in this field is ongoing, the rapid progress in gene editing has raised ethical concerns, regulatory framework, and public acceptance regarding its application in different animals. To address these concerns, involving community members in dairy farming programs can help make the approach less controversial compared to other gene drive designs.^117^

## Conclusions and future prospects

This review critically discusses current understandings of the carbon foot print of milk production, diet, and emissions from enteric fermentation in dairy cattle. It further explores the genetic mechanisms involved in CH_4_ emission traits and their implications for breeding approaches aimed at addressing or halting CH_4_ emissions from dairy cattle. As the effect of global climate change intensifies, this knowledge holds the potential to open up new avenues for mitigating the effects of CH_4_ from dairy cattle on global climate change. The literature indicates that genetic methods could be employed to breed dairy cows with reduced CH_4_ emissions, and incorporating traits, such as feed conversion efficiency into the current breeding goals. Given the complexity of identifying low-emitting cows and the tradeoffs involved, this review emphasizes the adoption of holistic breeding approaches. These include selective breeding, microbiome-driven breeding, breeding for feed efficiency, and breeding by genome editing to ensure sustainable dairy production in the face of global warming. The literature also underscores the importance of DNA methylation (DNAm) in shaping the microbial community in the rumen and suggests exploring methods, such as modifying the epigenetic landscape as an alternative approach to reduce CH_4_ emissions. The need for further research on genome-wide DNAm profiles for this purpose is highlighted. In summary, this review provides valuable insights for the dairy breeding industry, offering strategies to reduce CH_4_ emissions while maintaining milk production traits.
